# Targeting the CDK6 Dependence of Ph+ Acute Lymphoblastic Leukemia

**DOI:** 10.3390/genes12091355

**Published:** 2021-08-29

**Authors:** Patrizia Porazzi, Marco De Dominici, Joseph Salvino, Bruno Calabretta

**Affiliations:** 1 Department of Cancer Biology, Sidney Kimmel Cancer Center, Thomas Jefferson University, Philadelphia, PA 19107, USA; bruno.calabretta@jefferson.edu; 2 Department of Biochemistry and Molecular Genetics, University of Colorado Anschutz Medical Campus, Aurora, CO 80045, USA; MARCO.DEDOMINICI@CUANSCHUTZ.EDU; 3 The Wistar Institute, Philadelphia, PA 19104, USA; jsalvino@Wistar.org

**Keywords:** cell cycle, kinase, apoptosis, chemotherapy, proteolysis-targeting chimeras (PROTACs)

## Abstract

Ph+ ALL is a poor-prognosis leukemia subtype driven by the *BCR-ABL1* oncogene, either the p190- or the p210-BCR/ABL isoform in a 70:30 ratio. Tyrosine Kinase inhibitors (TKIs) are the drugs of choice in the therapy of Ph+ ALL. In combination with standard chemotherapy, TKIs have markedly improved the outcome of Ph+ ALL, in particular if this treatment is followed by bone marrow transplantation. However, resistance to TKIs develops with high frequency, causing leukemia relapse that results in <5-year overall survival. Thus, new therapies are needed to address relapsed/TKI-resistant Ph+ ALL. We have shown that expression of cell cycle regulatory kinase CDK6, but not of the highly related CDK4 kinase, is required for the proliferation and survival of Ph+ ALL cells. Comparison of leukemia suppression induced by treatment with the clinically-approved dual CDK4/6 inhibitor palbociclib versus CDK6 silencing revealed that the latter treatment was markedly more effective, probably reflecting inhibition of CDK6 kinase-independent effects. Thus, we developed CDK4/6-targeted proteolysis-targeting chimeras (PROTACs) that preferentially degrade CDK6 over CDK4. One compound termed PROTAC YX-2-107, which degrades CDK6 by recruiting the Cereblon ubiquitin ligase, markedly suppressed leukemia burden in mice injected with de novo or TKI-resistant Ph+ ALL. The effect of PROTAC YX-2-107 was comparable or superior to that of palbociclib. The development of CDK6-selective PROTACs represents an effective strategy to exploit the “CDK6 dependence” of Ph+ ALL cells while sparing a high proportion of normal hematopoietic progenitors that depend on both CDK6 and CDK6 for their survival. In combination with other agents, CDK6-selective PROTACs may be valuable components of chemotherapy-free protocols for the therapy of Ph+ ALL and other CDK6-dependent hematological malignancies.

## 1. Introduction

Acute lymphoblastic leukemia (ALL) is a hematological malignancy characterized by abnormal proliferation of progenitor cells of the lymphoid lineage. ALL displays a bi-modal age distribution with a first peak of incidence around 1 to 4 years and a second peak after 55 years of age [1]. B-ALL accounts for about 90% of pediatric and 75% of adult cases, while the remaining cases are of T-cell lineage [2]. 

Adult B-ALL has, in general, a dismal outcome with long-term cure rates that have only modestly improved over the past decades and remain less than 20%. In particular, elderly patients exhibit reduced tolerance for high-dose chemotherapy and a higher frequency of treatment-related mortality [3]. By contrast, infant and pediatric B-ALL cases have better cure rates [4,5]. B-ALL 5-year relative survival rate varies by age: For adults ages 20 and older, the rate is 38% for ALL; for youth ages 0–19 years, it is 89% for ALL. 

B-ALL is a heterogeneous disease with a complex molecular pathogenesis that differs in children and adults, and involves an array of genetic aberrations including mutations and chromosomal translocations of genes with essential roles in the cell cycle and lymphoid cell development. The mechanisms of the disease in children and adults depend, in most cases, on aberrant expression of transcription factors (TFs) or the constitutive activations of kinases that promote the acquisition of self-renewal, and are responsible for the enhanced proliferation and the differentiation arrest of lymphoid progenitor cells [6]. 

Some disease subtypes have a worse than average outcome and are known as “high-risk B-ALL”. In adults, the most frequent ‘high-risk’ subtype is the Philadelphia-positive (Ph+) ALL which is characterized by the reciprocal t(9;22) (q34;q11) translocation involving the *BCR* and the *ABL1* genes. The *BCR-ABL1* fusion genes generate the BCR-ABL chimeric protein that has constitutive tyrosine kinase activity and serves as the biological driver as well as the therapeutic target of the disease [7].

About a quarter of B-ALL patients do not present with prototypical mutations, chromosomal translocations or karyotypic abnormalities and as such were traditionally defined as B-others. More recently, transcriptome analyses revealed that a consistent fraction of these cases exhibit expression profiles similar to those of Ph+ ALL. This subtype was originally identified independently by two groups and was defined as Philadelphia (Ph)-like [8] or as BCR-ABL1-like [9]. Ph-like ALL constitutes 15–25% of total B-ALL, therefore accounting for the majority of B-other cases, is associated with poor-prognosis and its frequency increases with age in a manner similar to Ph+ ALL [10,11]. The Ph-like ALL is also a “high-risk” subtype with a <25% 5-year survival. 

Similarly to Ph+ ALL, Ph-like ALL patients show frequent alterations of *IKFZ1* and *CDKN2A*, both of which confer resistance to therapy and correlate with poor outcome. Molecularly, the vast majority, if not all cases of Ph-like ALL, harbor alterations in genes conferring de-regulated kinase-activity; in particular, translocations involving the *CRLF2* gene are observed in about half of the cases and generate fusion proteins or lead to increased *CRLF2* expression driven by the immunoglobulin heavy-chain (IgH) enhancer [12]. Other common translocations involve *ABL1*, *CSF1R*, *PDGFRB*, *EPOR* or *JAK2*. Frequent mutations are also observed in the *IL7R*, *FLT3*, *JAK1* and *JAK2* genes. Most of the aforementioned mutations activate signal transduction pathways that appear to converge on STAT5. Depending on the molecular abnormality driving specific subtypes of Ph-like ALL, pharmacological targeting of the activated signaling pathways may be a promising therapeutic option for Ph-like ALL. Indeed, TKI inhibitors such as Imatinib/Dasatinib, mTOR inhibitors and JAK inhibitors have been effective in pre-clinical studies and are currently under investigation in the clinic [10].

Many new drugs including TKIs, immune checkpoint inhibitors, cell cycle inhibitors, monoclonal antibodies targeting B-cell markers, pro-apoptotic and epigenetic agents are currently under investigation in the clinic. Use of these promising agents, alone or in combination with standard cytotoxic agents, has dramatically broadened the therapeutic opportunities for B-ALL patients, improving clinical outcomes. In this review, we focus on the therapeutic strategies for Ph+ ALL with a special emphasis on inhibitors of cell-cycle kinases.

## 2. Mechanisms of Transformation by the BCR-ABL Oncoprotein

**Figure 1:**
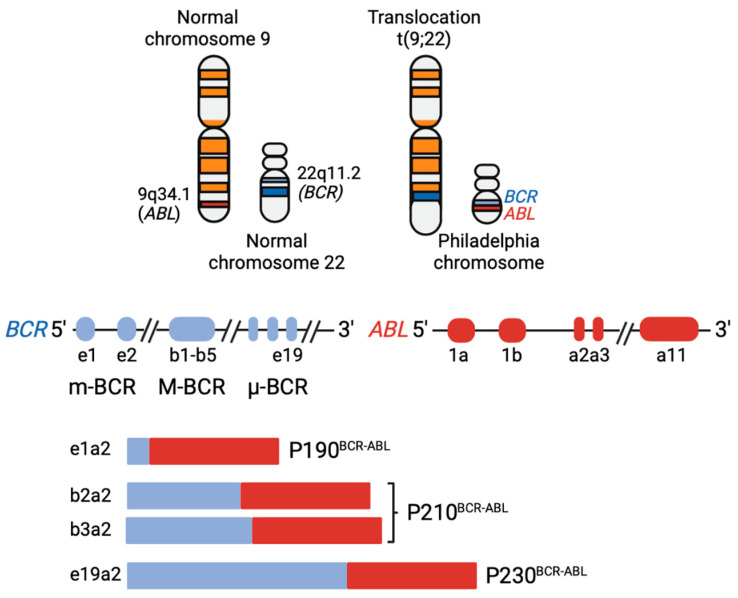
Representation of the Philadelphia chromosome translocation and BCR-ABL chimeric proteins. (Top) Translocation (9;22) and derivative Philadelphia-chromosome. (Bottom) *BCR* and *ABL* genes breakpoints and resulting fusion transcripts. *BCR* presents three breakpoints clusters: m-BCR, most associated to B-ALL; M-BCR, associated to CML; and μ-BCR, associated to chronic neutrophilic leukemia. *ABL* gene breakpoints usually occurs in between the first two exons (e1, e2), resulting in fusion proteins retaining the active tyrosine kinase domain of the *ABL* gene and different portions of the *BCR* gene. Image created with BioRender.com (2020).

Three BCR-ABL fusion proteins can derive from the rearranged genes of Philadelphia translocation, depending on the breakpoint on the *BCR* gene. When the break occurs in the major breakpoint of *BCR* (M-bcr) the 5′ of *BCR* up to exon 13 (also known as b2) or exon 14 (also known as b3) is fused to the *ABL1* gene in the first intron to generate the p210 BCR-ABL isoform. If the break occurs in the minor breakpoint of *BCR* (m-bcr) the first exon of *BCR* (e1) is fused to the first intron of the *ABL1* gene generating the p190 BCR-ABL isoform. Less frequently, the break can occur in the micro breakpoint of *BCR* (µ-bcr) which includes the *BCR* gene up to exon 19, generating the p230 BCR-ABL. The three *BCR-ABL1* fusion genes and the resulting chimeric proteins are associated with different leukemia subtypes (Figure 1). Chronic myeloid leukemia (CML) is almost exclusively associated with expression of the p210BCR-ABL isoform while in Ph+ B-ALL about three quarters of cases express the p190BCR-ABL isoform while the p210BCR-ABL isoform is expressed in the remaining cases. The p230BCR-ABL protein is only detected in a rare and mild myeloproliferative disorder characterized by pronounced neutrophilic differentiation known as chronic neutrophilic leukemia [13,14,15]. 

Expression of BCR-ABL is necessary for transformation of hematopoietic cells harboring the Philadelphia translocation. An extensive body of research has characterized the pathways activated by BCR-ABL. BCR-ABL can phosphorylate and activate its effectors directly but can also recruit protein complexes to activate specific signaling pathways. One of the most important targets of BCR-ABL are the Signal Transducer and Activation of Transcription family of proteins (STAT1, STAT2, STAT3, STAT4, STAT5a, STAT5b and STAT6). STATs are activated in hematopoietic cells by extracellular signals such as IL-3 and GM-CSF via the Janus Kinase proteins (JAK1, JAK2, JAK3 and Tyk2). Although BCR-ABL is capable of phosphorylating JAK1 and JAK2, it also phosphorylates and activates STAT proteins independently of JAKs activation [16,17,18]. STAT5 is clearly required for the development of CML since Stat5a/b deletion suppresses disease initiation and maintenance in mouse models of BCR-ABL1-dependent leukemia [19,20]. Moreover, a constitutively active mutant of STAT5 can induce leukemia when expressed in mouse hematopoietic stem cells [21]. The role of STAT5 is less clear in mouse models of Ph+ ALL, as it appears to be required for disease initiation but not for disease maintenance [22]. However, proliferation and colony formation of the Ph+ ALL line BV173 was markedly suppressed by STAT5 inhibition [23]. On the other hand JAK2, while dispensable for the activation of STAT5 in Ph+ cells, is important for the survival of BCR-ABL transformed cells. This is probably caused by its ability to induce, in a STAT5-independent manner MYC expression [24], since MYC plays an important role in BCR-ABL-dependent transformation [25], and maintenance of CML stem cells [26]. Recent work from our laboratory has also elucidated an alternative mechanism for MYC activation in Ph+ ALL which depends on STAT5-mediated activation of PIM1 [27]. Two additional pathways downstream of BCR-ABL have been thoroughly characterized, the PI3K-AKT pathway [28] and the RAS pathway leading to activation of ERK [29,30] or of c-JUN/JNK [31]. 

Compared to CML, Ph+ ALL is characterized by a much higher frequency of secondary mutations and chromosomal abnormalities, in particular translocations.

Secondary mutations in Ph+ ALL are, in part, associated with enhanced activity of the activation-induced cytidine deaminase *AICDA* (expressing AID) gene. Genes that appear to be mutated through this mechanism include the IgH variable (V) region genes, *BCL6*, and *CDKN2B* [32]. 

Another mechanism implicated in the genomic instability of BCR-ABL-expressing cells is their increased production of reactive oxygen species (ROS) [33]. Such an increase appears to be caused by the constitutive tyrosine kinase activity of BCR-ABL via a STAT5-dependent pathway and is associated with an increased frequency of BCR-ABL mutations [34,35]. Although there are no specific studies in Ph+ ALL, it is likely that activation of STAT5 mediated by the constitutively active BCR-ABL tyrosine kinase is also involved in a ROS-dependent mechanism of enhanced mutagenesis of the *BCR-ABL* gene.

In conclusion, the expression of BCR-ABL1 in hematopoietic stem cells and B-cell progenitors results in leukemia formation by several mechanisms converging in the promotion of cell proliferation, resistance to apoptosis and the acquisition of secondary mutations.

## 3. Therapy of Ph+ ALL

### 3.1. A. Chemotherapy

The Philadelphia chromosome is a marker of poor outcome in pediatric and adult B-ALL [36,37,38]. Prior to the development of BCR-ABL TKIs, Ph+ ALL had a dismal outcome with a 1-year survival of approximately 10% [39]. Today, the combination of new TKIs with chemotherapy, the improvement in stem cell transplantation and the availability of novel immunotherapeutic strategies has greatly improved patients’ outcome.

Chemotherapy in combination with TKIs is currently the standard treatment for Ph+ ALL. Typically, the treatment of de novo Ph+ ALL is divided in three phases: induction of remission, intensification/consolidation and maintenance [40,41]. The induction phase is the most aggressive phase of the therapeutic regimen and its aim is to reduce as much as possible patients’ leukemia burden and restoring normal hematopoiesis. Minimal residual disease (MRD) after the induction phase is typically monitored by high-sensitivity methods, which detect leukemic cells at a frequency of 1 × 10^−4^ or lower, such as flow cytometry analysis of leukemia-specific surface markers or qPCR to identify leukemia-specific *BCR-ABL1* transcripts. 

The achievement of MRD-negative status is the most important predictor of clinical outcome and can inform therapeutic decisions [42]. Another important factor is the localization of the disease in the central nervous system (CNS) that cannot be reached efficiently by most drugs and can serve as a reservoir of leukemic cells resulting in relapse. For this reasons, CNS-prophylaxis (intrathecal therapy or CNS irradiation) is usually included in the treatment protocol [43,44]. The induction phase includes agents such as vincristine (microtubule destabilizing agent), anthracyclines such as doxorubicin or daunorubicin (topoisomerase II inhibitors) and asparaginase (agent depleting asparagine which is essential for lymphoid cells), often conjugated with PEG to increase drug half-life. A common therapeutic protocol, named hyper-CVAD, consist of hyper-fractioned doses of cyclophosphamide (DNA-cross-linking agent), vincristine, doxorubicin and dexamethasone [45]. Glucocorticoids (dexamethasone and prednisone) are frequently used in association with chemotherapy. They function as ligands for the glucocorticoid receptor, which, upon activation, induces an endogenous immunosuppressive program leading to cell cycle arrest and apoptosis. Their advantage includes the ability to pass the blood-brain barrier providing activity in the CNS; on the other hand, being immunosuppressive agents, their use is associated with morbidity and mortality due to infections and may also cause psychiatric symptoms and bone toxicity [46]. 

After the induction phase, eligible patients in complete remission and with an appropriate donor undergo bone marrow transplantation. Bone marrow transplantation remains, to date, a curative treatment for young patients affected by Ph+ ALL, especially for those who achieve undetectable minimal residual disease (MRD) after induction therapy [47]. Maintenance therapy with tyrosine kinase inhibitors (TKIs) after transplantation is well tolerated and may reduce the risk if relapse, and is thus frequently incorporated in post-transplant regimens, despite a lack of consensus [48,49]. 

For Ph+ ALL patients who are not eligible for bone marrow transplantation, the consolidation/maintenance phase includes treatment with methotrexate (anti-folate agent) and mercaptopurine (inhibitor of de-novo nucleotide synthesis) with pulses of vincristine-dexamethasone [41].

### 3.2. B. BCR-ABL Tyrosine Kinase Inhibitors (TKIs)

The first specific inhibitor for the BCR-ABL tyrosine kinase was developed in 1996. imatinib (known originally as CGP-57148, STI-571 and subsequently as Glivec) was shown to inhibit BCR-ABL and the non-translocated ABL, as well as the PDGFRa/b and c-KIT but not other SRC-family proteins or other kinases, functioning as a competitive ATP binding inhibitor [50,51]. In vitro, imatinib markedly suppressed the viability of BCR-ABL expressing cells with no effect against normal cell lines and bone marrow cells [51]. In CML patients, imatinib induced a complete hematologic response (normalization of white blood cell count) in all cases and a major cytogenetic response (reduction in the number of Ph+ cells in the bone marrow) in 54% of patients [52]. Currently, treatment with imatinib is associated with >10-year survival and major cytogenetic response in approximately 33% of CML patients [7]. 

In patients with CML-myeloid blast crisis and CML-lymphoid blast crisis/Ph+ ALL, treatment with imatinib showed an early response with a marked reduction of circulating or bone marrow leukemic cells. However, in contrast to CML, the effect was transient with almost all patients relapsing within 6 months of treatment [53]. It was immediately apparent that imatinib resistance was associated, in most cases, with mutations in the tyrosine kinase domain of BCR-ABL and in some cases with BCR-ABL amplification [54]. In particular, the T315 mutation in the ABL kinase domain which blocks the access of imatinib to the ATP binding site was frequently observed. 

Second generation TKIs, including nilotinib and dasatinib, were subsequently developed. Nilotinib is structurally very similar to imatinib and has the same spectrum of activity toward tyrosine kinases. Dasatinib has several advantages over other TKIs: (i) it is about 300-fold more potent than imatinib; (ii) it is active against SRC-kinases; (iii) in contrast to imatinib and nilotinib, it binds BCR-ABL in the active conformation; and (iv) it is not a substrate of the P-glycoprotein (which mediate multi-drug resistance) [55]. Notably, dasatinib is the only TKI shown to pass the blood-brain barrier, potentially protecting against CNS relapse [56]. When tested in imatinib-resistant or intolerant CML-chronic phase patients, dasatinib induced a complete hematologic response and a major cytogenetic response in 92% of patients.

Responses were sustained in 95% of patients with a median follow-up of more than 12 months. In Ph+ ALL patients, 70% showed a complete hematologic response and a cytogenetic response. However, as seen with other TKIs, relapse was observed in all cases within 6 months [57]. The T315I mutation of BCR-ABL is the Achilles’ heel of TKIs since it is the most common secondary mutation and confers resistance to imatinib and all second-generation inhibitors [58]. Ponatinib, a third-generation BCR-ABL TKI, was introduced in 2013 and was shown to be active against the T315I BCR-ABL mutant. In the first clinical trial, ponatinib induced a complete hematologic response and a major cytogenetic response in 94% and 46% respectively, of CML-chronic phase patients who failed a previous therapy with a TKI and had the T315I mutation in 24% of the cases. With a median follow-up of 15 months, almost all patients remained in remission. Conversely, in Ph+ ALL, complete hematologic response and cytogenetic response were observed in 41% and 38% of patients respectively. However, almost all patients relapsed within 12 months [59]. In Ph+ ALL patients, ponatinib was directly compared to dasatinib as frontline therapy in a treatment regimen including hyper-CVAD and showed improved 3-year event-free survival and overall survival (83% for ponatinib vs 56% for dasatinib) [60]. By studying the mechanism of resistance, it became clear that no single, direct point mutation confers resistance to ponatinib; however, compound mutations or the subsequent mutation of isoleucine 315 into a methionine renders BCR-ABL insensitive to ponatinib treatment [61]. 

It is also apparent that mutations of BCR-ABL are more common in Ph+ ALL than in CML blasts which may explain the higher rates of treatment failure in Ph+ ALL. Interestingly, secondary BCR-ABL mutations in Ph+ ALL might be associated with aberrant expression of the *AICDA* gene which induces C to T transitions that could generate the T315I, E255K and Y253H mutations [58]. 

In summary, the chemotherapy/ponatinib combination represents an option for the treatment of Ph+ ALL, but the side effects of ponatinib, in particular cardiovascular toxicity, are significant [62]. A limitation of BCR-ABL targeting therapies is the development of BCR-ABL-independent resistance through poorly understood mechanisms. In the majority of cases, Ph+ ALL exhibits the deletion of the *IKZF1* gene [63], which encodes a TF that mediates pre-B-cell receptor signaling, inducing tumor suppression. The deletion of *IKZF1* is associated with more frequent and early-onset relapses [8,64]. Similarly, *CDKN2A* deletion is associated with poor response to therapy probably through a process of clonal selection during treatment with TKIs [65,66]. Another factor that confers resistance to TKIs treatment is aberrant expression of *BCL6*, a TF that represses the p53 pathway at multiple levels and has been shown to be induced by treatment with TKIs in Ph+ ALL [67]. In addition, an important role in TKI resistance is played by the microenvironment since the stromal support in vitro was capable of fostering the outgrowth of dasatinib-resistant Ph+ ALL cells [68]. Such growth-promoting effect by the microenvironment may be due to the expression of *CXCL12* and *IL-7* which, in turn, protect Ph+ ALL cells against the effects of TKIs [69]. 

### 3.3. C. Chemotherapy-Free Treatment

The current therapeutic regimen for Ph+ ALL, the combination of chemotherapy with TKIs, does not overcome the major hazard of each individual treatment which are toxicity and development of resistance mutations in the *BCR-ABL1* gene.

Chemotherapy-induced myelotoxicity leads to infections which are responsible for death of 10–20% of adult patients and of a higher percentage of the elderly ones. The majority of relapses in Ph+ ALL patients treated with chemotherapy and TKIs is caused by BCR-ABL mutations, in particular T315I. 

In an attempt to improve the outcome of Ph+ ALL, a chemotherapy-free approach consisting of dasatinib and corticosteroids followed by two cycles of blinatumomab, a bi-specific T-cell engager antibody that recruits T cells to CD19+ B-cells, was recently investigated in a phase 2 clinical trial of newly diagnosed Ph+ ALL patients [70]. This therapeutic approach led to complete remission and molecular response in 98 and 60% of the patients, respectively, with an 18-month overall survival in 95% of the patients. The clinical outcomes of this clinical trial are very encouraging, but its long-term benefits are unclear since relapses occurred in patients with *IKZF1* deletions and poor-prognosis secondary mutations (*CDKN2A* or *CDKN2B*). In addition, some of these patients exhibited the outgrowth of clonal leukemic populations carrying the T315I BCR-ABL mutation. 

The results of this clinical trial compare favorably to the ponatinib/chemotherapy combination trial which was associated with notable toxic effects [71]. However, a longer follow-up of a larger cohort of Ph+ ALL patients treated with this chemotherapy-free regimen, in particular those also receiving a bone marrow transplant as consolidation therapy, is needed to determine whether this approach will replace the chemotherapy/TKIs combination as standard of care for Ph+ ALL. 

**Figure 2:**
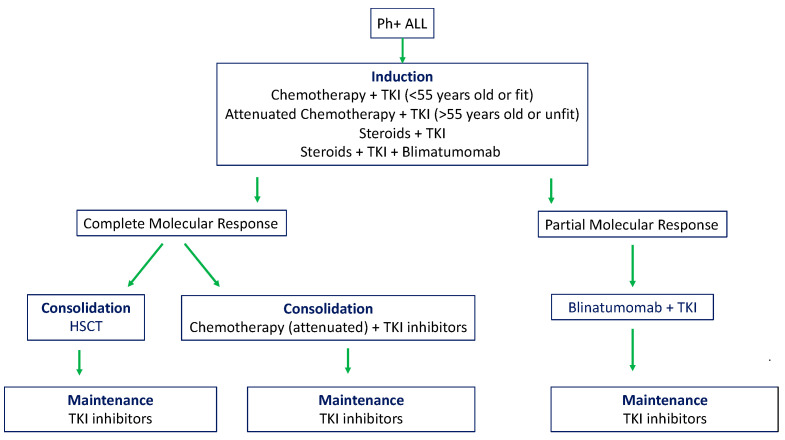
Treatment of adults with Philadelphia positive B-cell ALL. HSCT, hematopoietic stem cell transplant; TKI, tyrosine kinase inhibitor.

A summary of treatment regimens in adults with Ph+ ALL is presented in Figure 2.

## 4. Role of the MYB-CDK6 Pathway in Ph+ ALL Cells 

Several therapeutic options exist for patients with Ph+ ALL but the outcome for a significant portion of them, especially those with TKI-resistant disease, remains dismal. For this reason, we have investigated for many years the biology of Ph+ ALL, in search of vulnerabilities of leukemic cells that might be exploited therapeutically [72,73,74,75]. The transcription factor *MYB* (also known as *c-MYB*), and the MYB-CDK6 axis, have been thoroughly investigated by us and others and constitute a promising therapeutic target [76,77,78].

BCR-ABL-transformed B-cells are “addicted” to the expression of *MYB* [78], similar to BCR-ABL1-transformed myeloid cells, AML cells and a subset of T-ALL cells [79,80,81].

Upon assessing the changes in gene expression induced by MYB silencing, we noted that levels of *cyclin D3*, *CDK6* and *E2F*-regulated genes were markedly decreased in MYB-silenced Ph+ ALL cells [78]. Such changes suggest that MYB silencing impairs CDK6 activity, blocking E2F activation and S phase entry. Indeed, by selectively silencing the expression of either CDK6 or the related CDK4 kinase, we noted that the growth of Ph+ ALL cells was suppressed upon CDK6 but not CDK4 silencing. The precise mechanism responsible for the selective requirement of CDK6 by Ph+ ALL cells is unknown; however, CDK6 is predominantly localized in the nucleus, whereas CDK4 is exclusively localized in the cytoplasm of Ph+ ALL cells, possibly explaining why its expression is dispensable for Ph+ ALL growth [78].

Thus, targeting MYB or relevant MYB-regulated pathways that rely upon CDK6 activity might represent an attractive therapeutic strategy, especially for relapsed/TKI-resistant Ph+ ALL. 

In recent years, several candidate MYB inhibitors were identified including parthenolide, naphthol AS-E phosphate, and celastrol [82,83,84]. However, none of these compounds has been tested clinically and their growth-suppressive effects in BCR-ABL-expressing cells may not be entirely specific due to multiple mechanisms of action of these compounds [83,85,86,87].

MYB-dependent transcription and AML cell growth was also suppressed by a non-functional peptide that disrupts the interaction of MYB with TAF1, an essential component of the general transcriptional co-activating complexes TFIID and SAGA [88]. However, it is unclear if this is a viable therapeutic approach, given potential pleiotropic side effects induced by targeting a general transcriptional co-activator. 

*MYB* expression is required for the proliferation and survival of normal and leukemic hematopoietic cells [89,90] and the interaction of MYB with the p300 acetyltransferase protein is required for MYB-dependent transformation ex vivo [91] and for induction of AML by several oncogenes [92]. Thus, it has been hypothesized that blocking the MYB-p300 interaction may have therapeutic value for the treatment of MYB-dependent leukemia [77,93]. 

Several candidate compounds that disrupt this interaction were identified [84,94,95], but preliminary data of in vivo efficacy exist only in mice injected with MLL-rearranged AML cells and treated with a peptidomimetic inhibitor of the MYB-p300 interaction [95]. Thus, further assessment of the anti-leukemia effects of these inhibitors of MYB function is needed. 

Based on our findings that MYB silencing in Ph+ ALL cells induces a marked decrease in CDK6 expression/activity with no effects on CDK4 levels [78], we reasoned that targeting CDK6 with a CDK4/CDK6 inhibitor [96,97] may mimic key biological effects induced by MYB down-regulation. Indeed, pharmacological inhibition of CDK6 activity with the CDK4/6 inhibitor palbociclib suppressed growth of Ph+ ALL cells ex vivo and in mice [78]. 

The regulation of cell cycle progression in eukaryotic cells is accomplished by changes in the activity of cyclin-cyclin dependent kinases (CDK) complexes. While the expression of CDK proteins is relatively constant throughout the cell cycle, cyclins levels change in response to proliferative signals, resulting in the activation of different cyclin-CDK complexes in different phases of the cell cycle. The G1 to S phase progression through the so-called “restriction point” determines the commitment of cells to divide and is signaled by increased activity of CDK4/CDK6 in complex with D-type cyclins [98]. The primary role of CDK4/CDK6 is to phosphorylate and inhibit the Retinoblastoma (RB) protein and its homologs RBL1 and RBL2, promoting the activation of the E2F TFs, which is followed by CDK2 activation and the initiation of DNA replication [99]. The existence of multiple *CDK* and cyclin genes (13 and 25 respectively, although only 4 *CDK* and 10 *CYCLIN* genes are directly involved in cell cycle regulation) allows for the regulation of cell proliferation in specific contexts, while, at the same time, providing redundancy as suggested by the fact that single deletion of most *CDK* genes (except for *CDK1*) or cyclin genes has negligible effects in most cells. On the other hand, cancer cells are often characterized by mutations leading to hyperactivation of specific cyclin-CDK complexes, which might constitute potential candidates for targeted therapies [100]. Consistent with these ideas, the deletion of CDK6 caused only moderate reduction in the number of most hematopoietic cells, with decreased cellularity of the spleen and the thymus [101]. These effects were not associated with serious phenotypic abnormalities. Interestingly, even the combined deletion of CDK4 and CDK6 allowed the proliferation of mouse embryonic fibroblasts and development up to the late embryonic stage, at which point hematological defects became significant [101]. In hematopoietic stem cells, CDK6 expression regulates the time to exit from quiescence but is not necessary in cycling cells [102]. In addition, CDK6 has been suggested to be important under stress conditions (such as during transplantation) but not during normal hematopoiesis [103]. Conversely, CDK6 activity is often increased by various mechanisms in tumors, especially in hematological malignancies. CDK6 was found to be uniformly overexpressed in T-cell leukemia/lymphoma in the absence of gene amplification [104], and higher expression of CDK6 but not CDK4 was observed in B-ALL cells in comparison to normal bone marrow cells [105]. The *CDK6* locus is amplified in up to 23% of cases of T-cell lymphoma [106]. In addition, several studies found that in a fraction of B- and T-cell malignancies, aberrant RAG-induced recombination result in the t(2;7) translocation that juxtaposes the immunoglobulin light chain locus to the *CDK6* locus driving *CDK6* expression [107,108,109]. Moreover, *CDK6* is negatively regulated by miR-124 which is suppressed by promoter methylation in ALL cells and this event is associated with worse clinical outcome [110]. In BCR-ABL transformed cells, JUN activation prevents *CDK6* promoter methylation which is a limiting factor for leukemogenesis [111] and finally in 30% to 50% Ph+ ALL cases the *CDKN2A* locus is deleted, removing the inhibition of CDK6 by p16-INK4A [63,65]. Altogether, these studies suggest that in hematological malignancies, CDK6 has an important role in the regulation of cell proliferation. In agreement with this view, two studies showed that CDK6 but not CDK4 is required for the proliferation of MLL-rearranged leukemia, despite the fact that both kinases are expressed [105,112]. 

While CDK6 activity promotes cell cycle progression, multiple lines of evidence indicate that CDK6 exerts other activities, some of which kinase-independent, that may be important for the phenotype of leukemic cells. 

Firstly, CDK6 has been shown to block differentiation of myeloid cells [112,113] and this may be due to interaction with and inhibition of RUNX1 [114]; at the same time, CDK6 can phosphorylate and potentiate RUNX1 transactivation potential, promoting cell proliferation [115]. These findings suggest that CDK6 regulates RUNX1 by two mechanisms (physical interaction and phosphorylation) that might be alternatively utilized during differentiation and proliferation. In support for the importance of CDK6 activity in leukemia, an unbiased cDNA screen identified *CDK6* as a gene promoting imatinib resistance in v-ABL transformed cells. Furthermore, CDK6 was found to phosphorylate the transcription factor FOXM1 and prevent its ubiquitin-dependent degradation [110]. FOXM1 is a particularly interesting target because of its multiple oncogenic roles in different types of cancer [116] including B-ALL [117]. In addition to regulating transcription trough RB/E2F and FOXM1, CDK6 has been reported to act as a transcription (co)-factor (CDK6 lacks a DNA-binding motif). In this role, CDK6 regulates the expression of *EGR1* [103], *VEGF* and *p16INK4A* [111] and several genes of the p53 pathway [118]. Finally, CDK6 activity modulates the metabolism of T-ALL cells by phosphorylating and inhibiting 6-phosphofructokinase and pyruvate kinase M2, inducing a protective antioxidant function that prevents ROS-dependent cell death [119]. We and others have shown that inhibition of CDK6 activity is associated with changes in the transcription of various genes. While some of these effects are due to regulation of the activity/stability of transcription factors such as RB/E2F and FOXM1, studies from the Sexl laboratory have shown a direct transcriptional role for CDK6 in the regulation of genes important for hematopoiesis and leukemogenesis [103,120] as well as several regulators of p53 function [118]. Most of these genes were shown to be regulated in a kinase-independent manner. 

Palbociclib inhibits CDK6 activity but cannot suppress *CDK6* expression; in fact, we observed a compensatory CDK6 up-regulation after Palbociclib treatment [78]. This increase in CDK6 levels might be associated to enhanced kinase-independent transcriptional activation and might also result in resistance to palbociclib treatment, given that in clinical trials, palbociclib peak plasma concentration is not much higher than that necessary for effective inhibition of RB phosphorylation in vitro [121]. 

In summary, CDK6 does not function solely as a regulator of S-phase entry but can affect cancer cells, in particular different types of leukemic cells, in a way that might be exploited therapeutically.

## 5. Inhibiting CDK6 Expression by Proteolysis-Targeting Chimera (PROTACs) Suppresses Ph+ ALL 

Due to growing evidence suggesting that CDK6 has several pro-tumorigenic functions besides the regulation of the G1-S transition, agents that reduce CDK6 expression are expected to suppress leukemia growth through canonical (kinase-dependent) and non-canonical (kinase-independent) mechanisms. Moreover, such agents would also display fewer side effects compared to dual CDK4/6 inhibitors as normal hematopoietic progenitors rely on both CDK4 and CDK6 for their growth. 

Designing a CDK6-selective ATP competitive inhibitor devoid of activity toward CDK4 is challenging because the ATP binding domain (where typical small molecule inhibitors bind) of human CDK4 and CDK6 is virtually identical, except for a Glu21 of CDK6 that is replaced by Val14 in CDK4. Moreover, such compound would not inhibit the kinase-independent effects of CDK6.

Proteolysis-targeting chimeras (PROTACs) represent a new paradigm in pharmacology with the potential to be highly effective anti-cancer therapeutic agents. PROTACs are bi-functional molecules comprised of a targeting arm directed towards the protein of interest and an E3 ligase-recruiting arm connected by a linker; this allows the recruitment of various E3 ligases for optimal ubiquitination and proteasomal degradation of the protein of interest [122,123]. Compared to a traditional competitive inhibitor, a PROTAC requires less drug exposure due to its catalytic mechanism, where it binds a substrate, recruits an E3 ligase for ubiquitination/proteasomal degradation, and is released to repeat the cycle [124]. In contrast, a competitive inhibitor is required to bind continuously a non-degraded target protein in order to inhibit its activity, thus requiring 10–50 fold excess drug exposures over the IC_50_ to ensure effective inhibition [125]. 

Our group and others envisioned that selective CDK6-PROTACs could be developed due to differences in exposed lysine residues between CDK4 and CDK6, making it possible to identify CDK4/6-targeted PROTACs with preferential poly-ubiquitinatination and proteasomal degradation of one of the two related kinases. Therefore, discovery efforts were undertaken to identify PROTACs that would selectively degrade CDK6 over CDK4 [126,127,128]. 

We reported the development of novel CDK6-targeting PROTACs consisting of high-affinity small molecule ligands for CDK4/6, and for the E3 ubiquitin ligase Cereblon, joined by linkers of different structure and/or size [129].

In order to generate a suitable CDK6 targeting PROTAC a potent CDK6 ligand was conjugated to an E3 ligase recruiting molecule, such as a thalidomide derivative, using a suitable linker. We considered Palbociclib as a CDK6 targeting ligand since it potently inhibits CDK6, it is an FDA approved CDK4/6 kinase inhibitor, and the X-ray crystal structure of CDK6 in complex with Palbociclib is available (pdb ID 2euf) [130]. Palbociclib’s piperazine moiety was suggested as optimal region to install a linker since the X-ray structure reveals this moiety is protruding into solvent. Surprisingly this region was sensitive to linker attachment and a variety of linkers and linkers with various E3 ligase recruiting ligands were evaluated until we identified a Palbociclib conjugate that potently inhibited CDK6 and preferentially degraded CDK6 compared side by side to CDK4 by Western Blot analysis. The Cereblon-recruiting PROTAC YX-2-107 was identified as a potent inhibitor of in vitro CDK4 or CDK6 kinase activity (IC50 = 0.69 and 4.4 nM, respectively) comparable to Palbociclib (IC50 = 9.5 nM) as well as a selective CDK6 degrader in Ph+ BV173 ALL cells with a degradation constant, DC50, of ~4 nM, based on densitometric analysis of CDK6 band intensities over a range of YX-2-107 concentrations [126]. 

In particular, one PROTAC termed YX-2-107 not only degraded rapidly and preferentially CDK6 over CDK4, but also inhibited CDK4/6 enzymatic activity in vitro with IC50 comparable to that of palbociclib (Figure 1). Of greater importance, PROTAC YX-2-107 exhibited kinase-dependent and independent effects in Ph+ cell lines, and suppressed patient-derived Ph+ ALL growth in vivo, using immunodeficient (NSG) mice.

**Figure 3:**
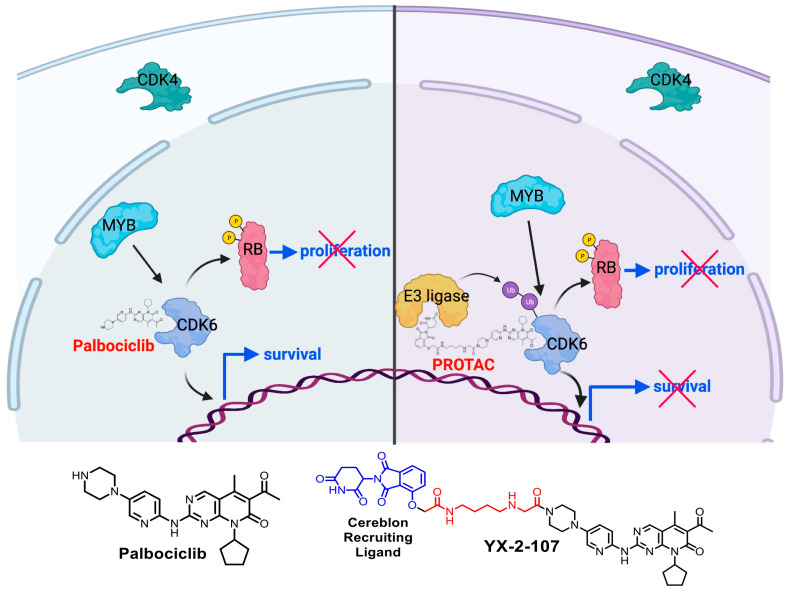
Inhibition of CDK6 activity in Ph+ ALL. Growth of Ph^+^ ALL cell lines and primary cells is dependent on MYB-dependent transcriptional regulation of CDK6. CDK6 is a key cell cycle regulator predominantly localized in the nucleus of Ph+ ALL cells. Pharmacologic inhibition of CDK6 can be achieved with the kinase inhibitor Palbociclib (left). This results in suppression of leukemic cell proliferation but does not affect the kinase-independent function of CDK6. CDK6-targeted proteolysis-targeting chimeras (PROTACs; YX-2-107, right) are molecules comprising a ligand for the protein of interest and an E3 ligase–recruiting ligand that are connected by a linker, allowing recruitment of E3 ligases for optimal ubiquitination and proteasomal degradation. YX-2-107 promotes the rapid and preferential degradation of CDK6 over CDK4 in Ph+ ALL cells, suppressing the proliferation and survival of de novo or TKI-resistant primary Ph+ ALL cells. CDK6 degradation suppresses Ph^+^ ALL more effectively than CDK4/6 inhibition, due to suppression of kinase-dependent and independent effects of CDK6. Image created with BioRender.com (2020).

Initial evidence in support of CDK6 kinase-independent effects in Ph+ ALL came from the comparison of the effects induced by CDK6-silencing vs CDK6 inhibition [126]. We observed that CDK6-silenced Ph+ ALL cells are more susceptible to apoptosis and exhibit a slower disease progression in NSG mice than the palbociclib-treated counterparts, possibly as consequence of lower expression of genes involved in cell survival, chromatin remodeling and mitochondrial metabolic pathways for energy production observed in CDK6-silenced cells. Interestingly, the expression of *CDK6* and *HDAC1* was highly correlated in a dataset of 122 Ph+ ALL patient’s samples, suggesting that the CDK6-HDAC1 pathway may be critical for the growth suppression/apoptosis of CDK6-silenced Ph+ ALL cells. Together, these features of CDK6-silenced Ph+ cells support the idea that CDK6 degraders might be more effective therapeutic agents than CDK4/6 enzymatic inhibitors because the latter drugs are predominantly cytostatic whereas CDK6 degraders are also cytotoxic (Figure 3).

While treatment of Ph+ ALL cells with PROTAC YX-2-107 promotes the preferential degradation of CDK6 over CDK4 in Ph+ ALL cells, treatment with palbociclib induced an increase in the levels of CDK6 that is likely to contribute to the development of palbociclib resistance [129,131]. Moreover, proteomic analysis of Ph+ BV173 cells treated with PROTAC YX-2-107 revealed that this compound is highly specific, since CDK6 was the only significantly downregulated protein detected out of 3682 examined. Similar to palbociclib, YX-2-107 exhibited a relatively long half-life when incubated in mouse liver microsomes, which typically correlates with potent in vivo activity. However, YX-2-107 had a half-life of 1 h in a mouse PK study when administered by IP, suggesting that further improvement in PK is warranted. A 48-h YX-2-107 treatment of NSG mice with advanced Ph+ ALL was sufficient to markedly decrease the proportion of S phase cells in the bone marrow, suppress the expression of the CDK4/6 substrates phospho-RB and FOXM1, and induce the preferential degradation of CDK6 over CDK4. A long-term (2–3 weeks) treatment of NSG mice injected with de novo or TKI-resistant primary Ph+ ALL induced a marked suppression of peripheral blood leukemia load that was comparable or even superior to that induced by treatment with palbociclib. PROTAC YX-2-107 was also highly effective in suppressing the growth of a TKI-resistant Ph+ ALL in human cytokine-expressing NRG mice [126]. This finding suggests that neither pharmacological resistance to TKIs, nor exposure to cytokines present in the human microenvironment impairs the therapeutic effects of PROTAC-based CDK6 degraders. 

These in vivo effects represent the first demonstration of therapeutic efficacy by a PROTAC that targets an oncogenic protein which exerts kinase-dependent and independent effects. 

## 6. Future Approaches for CDK6-Targeted Therapies in Ph+ ALL

While suppressing *CDK6* expression alone exerts anti-leukemia effects in our pre-clinical studies in mice injected with primary Ph+ ALL cells, treatment with PROTAC YX-2-107 may not achieve a long-lasting therapeutic response. To address this potential problem, we have generated derivatives of PROTAC YX-2-107 that are more potent in inhibiting S phase and in inducing CDK6 degradation or possess better metabolic stability. Further development is expected to yield second-generation PROTACs combining superior ex vivo activities (inhibition of CDK6-regulated S phase; CDK6 degradation) with improved pharmacokinetic properties that might establish these drugs as *bona fide* anti-cancer therapeutics.

Other compounds may collaborate with CDK6-degrading agents in suppressing more profoundly Ph+ ALL by targeting additional pathways that are essential for the growth of these cells. For example, BET inhibitors are known to suppress the transcription of several genes (e.g., *CDK6* itself, *BCL2* and *MYC*) which are all required for the growth of Ph+ ALL cells. Incidentally, the expression of these three genes is transcriptionally regulated by MYB which is also a target of BET inhibitors in leukemic cells due the involvement of “superenhancers” in regulating its transcription [132]. 

We have also shown that CDK6 inhibition result in increased levels of apoptosis when combined with the pan-BCL2 inhibitor Sabutoclax, in agreement with the reported activity of CDK6 in regulating several genes with a role in cell survival. 

An area that merits further investigation is the search for agents that result in synergistic effects when combined with CDK6 inhibition. It was previously shown that *CDK6* expression confers resistance to Imatinib treatment [133]. It would be interesting to evaluate whether CDK6 inhibitors can cooperate with TKIs in inducing a more profound clearance of leukemic cells and possibly increasing the likelihood of achieving MRD-negative status, which is a powerful predictor of response to therapy in ALL [42,134]. 

Another potential approach would be to combine a CDK6-selective PROTAC with dexamethasone since the latter was shown to regulate the expression of *MYB*, *BCL2* and *BIM* in preclinical models of ALL [135]. Interestingly, two recent studies have shown synergistic effects for the combination of Ribociclib (another CDK4/6 inhibitor) and dexamethasone in Ph-negative B-ALL and T-ALL respectively [136,137]. 

Since the “MYB addiction” of Ph+ ALL is not only due to *CDK6* expression but also to other MYB-regulated genes such as *BCL2*, an alternative therapeutic strategy for Ph+ ALL is to combine a CDK6-selective PROTAC with a selective BCL2 antagonist like Venetoclax. This approach could be personalized by pre-selecting Ph+ ALL patients for whom there is evidence that their leukemic cells undergo apoptosis ex vivo upon treatment with Venetoclax.

In conclusion, these potential CDK6-based therapies may provide additional opportunities for an effective and less toxic therapeutic regimen for Ph+ ALL, especially the TKI-resistant/refractory stage.

## Data Availability

Not Applicable.
